# Epidemiology of complications of male circumcision in Ibadan, Nigeria

**DOI:** 10.1186/1471-2490-6-21

**Published:** 2006-08-25

**Authors:** Linus I Okeke, Adanze A Asinobi, Odunayo S Ikuerowo

**Affiliations:** 1 Surgery Department, College of Medicine, University of Ibadan/University College Hospital, Ibadan, Nigeria; 2 Pediatrics Department, College of Medicine, University of Ibadan/University college Hospital, Ibadan, Nigeria

## Abstract

**Background:**

The number of infants managed for neonatal circumcision injuries in our unit has been on the increase over the past 16 years. In our search for the sources and reasons for these injuries, we were unable to identify any previous studies of circumcision injuries from our environment. We therefore decided to carry out this study in order to shed some light on this growing problem.

**Methods:**

The patients were made up of 370 consecutive consented children attending our infant welfare clinic for immunization over a period of 3 months. Information on their demographic data, their age at circumcision, where, why and who circumcised them was obtained from their mothers. They were clinically examined for the presence and type of complications of circumcision.

**Results:**

Our circumcision rate was 87%. Neonatal circumcision had been performed in 270 (83.9%) of the children. Two hundred and fifty nine (80.7%) were performed in hospitals. The operation was done by nurses in 180 (55.9%), doctors in 113 (35.1%) and by the traditional circumcisionist in 29 (9%) of the children. Complications of circumcision occurred in 65 [20.2%] of the children. Of those who sustained these complications, 35 (53.8%) had redundant foreskin, 16 (24.6%) sustained excessive loss of foreskin, 11 (16.9%) had skin bridges, 2 (3.1%) sustained amputation of the glans penis and 1 (1.5%) had a buried penis. One of the two children who had amputation of the glans also had severe hemorrhage and was transfused. Even though the complications tended to be more likely with nurses than with doctors or traditional circumcisionists, this did not reach statistical significance (p = 0.051).

**Conclusion:**

We have a very high rate of complications of circumcision of 20.2%. We suggest that training workshops should be organized to adequately retrain all practitioners of circumcision on the safe methods available.

## Background

About 25% of the total world male population is circumcised and circumcision remains one of the oldest and commonest operations performed all over the world [1-4]. The complication rates of the procedure range between 0.19% and 3.1% [1,5-7]. The Fetus and Newborn Committee of the Canadian Pediatric Society and the American Academy of Pediatrics extensively considered the costs, complications and the presumed advantages of neonatal circumcision and recommended that circumcision of the newborn should not be performed routinely [8,9]. Despite this and many other similar recommendations, the procedure has continued to be performed even to a greater degree with virtually all children undergoing circumcision in the neonatal period in some communities [1]. In the last 16 years, we have received and managed a good number of children with circumcision injuries referred to our center. However, we were unable to identify any literature addressing this subject from our environment, hence our decision to carry out this study.

## Methods

Three hundred and seventy consecutive male infants attending the infant welfare clinic for immunization were studied over a period of 3 months between 1^st ^June and 31^st ^August 2005. The study was explained to their mothers who then gave their verbal consent. The mothers were interviewed to determine their religion and social status, the age of the children, circumcision status, their age at circumcision, where, why and who performed the operation. The penises of the children were examined to confirm their circumcision status, and determine the presence and type of any complication. Those who were not circumcised at the time of their enrolment into the study were followed up to the end of the study and their records amended if their circumcision status changed. Ethical clearance for this study was obtained from the Ethical Committee of the Department of Surgery, College of Medicine, University of Ibadan.

### Statistical analysis

The data was analysed using the SPSS 11.0 statistical software for Windows. Paired t-tests were used to evaluate for statistical significance and p-values ≤ 0.05 were considered significant.

## Results

We studied 370 male children over a 3 month period. The age of the patients ranged from 8 days to 13 months, with 357 (96.5%) being infants. Three hundred and twenty two were circumcised, giving a circumcision rate of 87%. There was no correlation between the circumcision status and the social status of the parents (Pearsons correlation .023, p = .682). Of the 322 children who were circumcised, 270 (83.9%) were done within the first month of life. Two hundred and sixty (80.7%) and 62 (19.3%) of the circumcisions were done in hospitals and at home respectively. None of the children was circumcised for a medical reason. The operation was performed by nurses in 180 (55.9%), doctors in 113 (35.1%) and by the traditional circumcisionist in 29 (9%) of the children. Complications occurred in 65 children [20.2%]. Of those who sustained these complications, 35 (53.8%) had redundant foreskin, 16 (24.6%) sustained excessive loss of foreskin, 11 (16.9%) had skin bridges, 2 (3.1%) sustained amputation of the glans penis while 1 (1.5%) had a buried penis (table 1). One of the two children who sustained amputation of their glans penis also had severe hemorrhage and had to be transfused with blood. The complications tended to be more likely with nurses than with doctors or traditional circumcisionists, but this did not reach statistical significance (p = 0.051).

## Discussions and conclusions

Our circumcision rate was 87%. This is much higher than the world average circumcision rate of between 25% and 33.3% [4,10] but less than the rate in Israel where virtually every male is circumcised [1]. Neonatal circumcision as we practice it is quite common worldwide. While the people of pacific origin prefer their children to be circumcised between the ages of 6 and 10 years [11], it is done in adolescence as an initiation rite to manhood [12] among the Xhosa tribe in Eastern Cape, South Africa. The majority of our cases were done in the hospitals with only 19.3% being done at home. In certain areas of the world, virtually all their circumcisions are done outside the hospitals [1,12] especially in such communities where it is done as part of initiation to manhood [12]. Like most other centers, the indication for circumcision in our environment remains largely non-medical, cutting across social, ethnic and religious barriers. Nurses performed a greater proportion of our cases but it is not unusual to have none-doctors perform these procedures [1,11,12]. We found a 20.2% complication rate. This is an unacceptably high rate compared to the figures from the rest of the world, which range between 0.19% and 3.1% [1,5-7]. Redundant foreskin constituted over half of the cases. This is in agreement with the findings of Ben Chaim et al in Israel [1] and Yegane et al in Iran [15]. Neonatal circumcision as it is practiced here is supposed to be easier and associated with less complications compared to adolescent or adult circumcision [13]. Our complications tended to be more likely with nurses than with doctors or traditional circumcisionists, but this did not reach statistical significance. Only in 57 patients were the mothers certain about the method of circumcision used for their children. As a result, the complication rates associated with the different methods of circumcision could not be computed. We believe that this unacceptably high complication rate of neonatal circumcision could be brought down by organizing training workshops to adequately retrain all practitioners of circumcision on the safe methods available [14] and how to use them.

## Competing interests

The author(s) declare that they have no competing interests.

## Authors' contributions

1. LIO: made substantial contributions to conception and design, analysis and interpretation of data; drafting the manuscript, revising it critically for important intellectual content; and gave final approval of the version to be published.

2. AAA: made substantial contributions to design, data collection, and manuscript revision for important intellectual content and gave final approval of the version to be published.

3. OSI: made substantial contributions to design, data collection, analysis, manuscript revision for important intellectual content and gave final approval of the version to be published.

All authors read and approved the final manuscript.

## Pre-publication history

The pre-publication history for this paper can be accessed here:


